# Gene polymorphism of MTHFR rs1801133 and susceptibility to childhood leukemia in Chinese population

**DOI:** 10.5937/jomb0-41672

**Published:** 2023-10-27

**Authors:** Yin Peng, Chengjun Wang, Lin Wu

**Affiliations:** 1 Anhui Provincial Children's Hospital, Department of Pediatric Nephrology, Hefei, China; 2 Anhui Provincial Children's Hospital, Department of Pediatric hematologic Oncology, Hefei, China

**Keywords:** MTHFR rs1801133, polymorphism, childhood leukemia, genetic susceptibility, MTHFR rs1801133, polimorfizam, leukemija u detinjstvu, genetska podložnost

## Abstract

**Background:**

The purpose of this study is to investigate the genotype and allele distribution of MTHFR rs1801133 in the Chinese population, and to analyze the relationship between gene polymorphism of MTHFR rs1801133 and risk of childhood leukemia.

**Methods:**

Blood samples and clinical data of childhood leukemia cases (n=1132) and age-matched healthy controls (n=1053) were collected. Genotypes and allele distribution of MTHFR rs1801133 were detected by PCR-RFLP. Logistic regression model was generated to analyze the relation between MTHFR rs1801133 and susceptibility to childhood leukemia and the chemotherapy response.

**Results:**

Age, sex, BMI and family history of tumor were comparable between childhood leukemia cases and healthy controls. Genotypes and allele distribution of MTHFR rs1801133 were remarkably correlated to the risk of childhood leukemia. Genotype risk of MTHFR rs1801133 was parallel to the susceptibility to childhood leukemia. Specifically, compared with people carrying AA allele of MTHFR rs1801133, higher risk of childhood leukemia may occur in people carrying AG+GG allele of MTHFR rs1801133 with a younger age (<15 years) or complete remission from chemotherapy.

**Conclusions:**

MTHFR rs1801133 gene polymorphism has a significant correlation with childhood leukemia. It is an important genetic susceptibility gene of childhood leukemia. The reliability of the results requires to be further validated by the high-quality research involving a large sample size in multi-center hospitals.

## Abstract (sr)

**Uvod:**

Svrha ove studije je da se ispita distribucija genotipa i alela MTHFR rs1801133 u kineskoj populaciji i da se analizira odnos između polimorfizma gena MTHFR rs1801133 i rizika od leukemije u detinjstvu.

**Metode:**

Prikupljeni su uzorci krvi i klinički podaci slučajeva leukemije u detinjstvu (n = 1132) i zdravih kontrola odgovarajućeg uzrasta (n = 1053). Genotipovi i distribucija alela MTHFR rs1801133 detektovani su PCR-RFLP Model logističke regresije je generisan da analizira odnos između MTHFR rs1801133 i osetljivosti na leukemiju u detinjstvu i odgovora na hemoterapiju.

**Rezultati:**

Starost, pol, BMI i porodična istorija tumora bili su uporedivi između slučajeva leukemije u detinjstvu i zdravih kontrola. Genotipovi i distribucija alela MTHFR rs1801133 bili su u značajnoj korelaciji sa rizikom od leukemije u detinjstvu. Rizik od genotipa MTHFR rs1801133 bio je paralelan sa osetljivošću na leukemiju u detinjstvu. Konkretno, u poređenju sa ljudima koji nose AA alel MTHFR rs1801133, veći rizik od leukemije u detinjstvu može se javiti kod ljudi koji nose AG+GG alel MTHFR rs1801133 mlađe starosti (<15 godina) ili potpune remisije od hemoterapije.

**Zaključak:**

Polimorfizam gena MTHFR rs1801133 ima značajnu korelaciju sa dečjom leukemijom. To je važan gen genetske osetljivosti na dečju leukemiju. Pouzdanost rezultata treba da bude dodatno potvrđena visokokvalitetnim istraživanjem koje uključuje veliki uzorak u bolnicama sa više centara.

## Introduction

Leukemia is a malignant disease attacking the hematopoietic system. It is the mostly diagnosed tumor in children. Based on disease course and cell differentiation, leukemia is classified to acute and chronic type. Acute leukemia (AL) covers about 95% of childhood leukemia [1] [2] [3]. Classified by malignant cell origins, AL is subgrouped to acute myeloid leukemia (AML) and acute lymphocytic leukemia (ALL). The incidence of ALL (3.4– 4.1/100,000) is 5 times that of AML [4] [5]. At present, chemotherapy is preferred to childhood ALL, and its 5-year survival is up to 85% [3] [6]. Even for the standard risk group patients, the 5-year progression-free survival can reach 93%. Nevertheless, chemotherapyinduced adverse effects and drug toxins seriously affect life quality of childhood leukemia patients [7] [8] [9].

The protein encoded by MHTFR (NADPH) is a key enzyme of folate metabolism. NAPDH catalyzes the conversion of 5, 10-methylenetetrahydrofolate to 5-methyltetrahydrofolate, a co-substrate for homocysteine (hCY) remethylation to methionine [10] [11]. The mutation of MTHFR gene leads to increased plasma concentration of hCY and DNA hypomethylation, which further affect the production of folic acid and the synthesis of DNA [11]. There are 14 mutations in the coding region of MTHFR gene, of which rs1801133 is the most common single nucleotide polymorphism (SNP) [12] [13]. Gene polymorphism of MTHFR in the development of childhood leukemia is rarely reported [14] [15]. In this case-control study, we aim to uncover the relation between MTHFR rs1801133 and susceptibility to childhood leukemia in Chinese population.

## Materials and methods

### Study population

Leukemia children (n=1132) and age-matched healthy controls (n=1053) were randomly recruited in Anhui Provincial Children’s Hospital. Eligible leukemia children did not have other malignancies or received pre-chemotherapy. Childhood leukemia was independently diagnosed by two pathologists based on clinical symptoms, physical, imaging and histological examinations.

Healthy controls were recruited from whom participated in physical examinations during the same period. They did not have blood connections to other participants. Family history of cancer was defined as the presence of cancer in any first-degree relatives (parents, siblings or offspring). This study was approved by the institutional review committee of our hospital. All participants and their parents were informed consent.

### PCR-RFLP (polymerase chain reaction - restriction fragment length polymorphism)

Genomic DNA was isolated and purified from peripheral blood lymphocytes by proteinase K digestion and phenol-chloroform method. MTHFR rs1801133 and its alleles were detected using PCR-RFLP (Applied Biosystems, Foster City, CA, USA). SNP primers were amplified at 95°C for 10 min, followed by 45 cycles at 95°C for 15 s and 60°C for 1 min. PCR products were cleaved by BccI, loaded on 1% DNA agarose gel containing C_21_H_20_BrN_3_, and analyzed.

### Statistical analysis

Statistical Product and Service Solutions (SPSS) 22.0 (IBM, Armonk, NY, USA) was utilized for statistical analysis. Enumeration data were expressed as frequency (%). The HWE of control genotype distribution, and comparison of enumeration data were evaluated using the x^2^ test. Risk factors of childhood leukemia were assessed by Logistic regression test, and results were expressed as OR and 95% CI. *P*<0.05 considered as statistically significant.

## Results

### Characteristics of childhood leukemia cases and healthy controls

A total of 1132 eligible leukemia children and 1053 age-matched healthy controls were recruited. By analyzing their clinical data, it is shown that age, sex, BMI and family history of tumor were comparable between childhood leukemia cases and healthy controls (*P*>0.05, Table 1).

**Table 1:** Distribution of selected variables between the childhood leukemia cases and the control subjects. ^*^Student’s t-test for age and BMI distributions between cases and controls; two sided x^2^ test for other selected variables between cases and controls.

Variables	Cases (n = 1132)		Controls (n = 1053)		P -value^*^
	N	%	N	%	
Age (mean ± SD), years	16.7±7.0		17.8±8.3		0.176
<15	893	78.89	875	83.10	
≥15	239	21.11	178	16.90	
Sex					
Male	442	39.05	513	48.72	0.072
Female	690	60.95	540	51.28	
BMI (mean ± SD), kg/m^2^	19.1±2.4		19.9±4.1		0.073
<24	730	64.49	802	76.16	
≥24	402	35.51	251	23.84	
Family					
No	880	77.74	835	79.30	0.208
Yes	252	22.26	218	20.70	

### Association between childhood leukemia risk and MTHFR rs1801133

Genotypes and allele distribution of MTHFR rs1801133 were detected to be remarkably correlated to the risk of childhood leukemia (Table 2). In particular, a higher susceptibility to childhood leukemia in people carrying GG (*P*=0.005, OR=1.56 (1.15–2.13)) or AG+GG allele (*P*=0.032, OR=1.17(1.01–1.35)) was identified in comparison to whom carrying AA allele. G allele of MTHFR rs1801133 (*P*=0.006, OR=1.12 (1.06–2.30)) predicted a higher risk of suffering from childhood leukemia than A allele.

**Table 2:** The basic information of the genotyped polymorphisms in MTHFR rs1801133 Polymorphism associated with the risk of Childhood leukemia. ^*^Adjusted for age, sex, BMI, smoking status, drinking status, diabetes and hypertension in logistic regression model.<br>CI, confidence interval; OR, odds ratio.

Polymorphisms	Cases (n = 1132)		Controls (n = 1053)		P^*^	Adjusted OR (95% CI)^*^
	N	%	N	%		
rs1944129						
AA	624	55.1	692	65.7		1.00 (reference)
AG	357	31.5	300	28.5	0.145	1.12(0.96–1.30)
GG	151	13.4	61	5.8	0.005	1.56(1.15–2.13)
AG+GG	508	44.9	361	34.3	0.032	1.17(1.01–1.35)
A allele	1605	74.1	1684	80.0		1.00 (reference)
G allele	659	25.9	422	20.0	0.006	1.12(1.06–2.30)

### Association between childhood leukemia pathology and MTHFR rs1801133

Logistic regression model was generated to analyze the relation between MTHFR rs1801133 and susceptibility to childhood leukemia and the pathology (Table 3, Table 4). Compared with people carrying AA allele of MTHFR rs1801133, a higher risk of childhood leukemia may occur in people carrying AG+GG allele of MTHFR rs1801133 with a younger age (<15 years; OR=1.23, 95%CI=1.09–1.97, *P*=0.014) or complete remission (OR=1.58, 95%CI=1.29–2.39, *P*=0.004).

**Table 3:** Association between MTHFR rs1801133 Polymorphism and Childhood leukemia in stratified analysis. ^*^Two-sided x^2^ test for number of risk alleles in cases and controls; 95% CI: 95% confidence.<br>Adjusted for age, pack-years of smoking, drinking status, and family history of cancer in logistic regression model.

Variables	Risk allele				P^*^	Adjusted OR (95% CI)^*^
	AA		AG+GG			
	Case n,%)	Control (n,%)	Case (n,%)	Control (n,%)		
Age						
<15	416 (66.6)	341 (49.3)	477 (93.9)	234 (64.8)	0.014	1.23 (1.09–1.97)
15	208 (33.4)	351 (50.7)	31 (6.1)	127 (35.2)	0.068	1.09 (1.18–1.64)
Sex						
Male	350 (56.1)	332 (48.0)	292 (57.5)	181 (50.1)	0.614	1.42 (0.77–1.86)
Female	274 (43.9)	360 (52.0)	216 (42.5)	180 (49.9)	0.508	1.11 (0.87–2.11)
BMI						
<24	406 (65.1)	483 (69.8)	414 (81.5)	279 (77.29)	0.429	1.19 (1.23–2.12)
24	218 (34.9)	209 (30.2)	94 (18.5)	82 (22.71)	0.333	1.02 (1.13–1.99)
Family						
No	590 (94.6)	656 (94.8)	492 (96.9)	319 (88.4)	0.561	1.34 (0.73–2.58)
Yes	34 (5.4)	36 (5.2)	16 (3.1)	42 (11.6)	0.714	1.65 (0.89–2.08)

**Table 4:** Association between MTHFR rs1801133 Polymorphism and clinicopathologic characteristics of Childhood leukemia. ^*^Adjusted for age, sex, BMI, family in logistic regression model. CI, confidence interval; OR, odds ratio.<br>CR, complete remission; PR, partial remission; NR, no remission

Variables	Risk allele				P^*^	Adjusted OR (95% CI)^*^
	AA		AG+GG			
	N	%	N	%		
Leukemia	624	100.0	508	100.0		1.00 (reference)
CR	467	74.8	322	63.4	0.004	1.58 (1.29–2.39)
PR	112	17.9	68	13.4	0.053	1.38 (0.79–1.98)
NR	45	7.2	118	23.2	0.097	0.98 (0.69–1.49)

## Discussion

ALL originates from lymphocyte progenitor cells. It is the most common hematological malignant disease in children. Malignantly proliferated immature hematopoietic stem cells are accumulated in peripheral blood and bone marrow, leading to suppression of hemopoietic function. In the meantime, malignant hematopoietic stem cells can infiltrate the liver, spleen, lymph nodes and other organs, resulting in hemorrhage, anemia, infection, and organ involvement manifestations [1] [2] [3]. Childhood leukemia mainly affects children in 2–5 years, especially male children. It is generally considered that chemical, environmental, and genetic factors are risks of childhood leukemia. In addition, the two-hit hypothesis has been widely recognized. Chromosomal changes occur in the fetus or at birth (first hit). After birth, gene deficiency or mutations caused by chemical or environmental factors further drive the carcinogenesis of childhood leukemia (second hit) (3–6). Chemotherapy-induced adverse effects are huge obstacles in the clinical treatment of childhood leukemia [7]. With the rapid development of genome-wide association studies, SNPs are emerged to have a certain correlation with the incidence of childhood leukemia [8] [9].

MTHFR is a key enzyme affecting Hcy metabolism. MTHFR rs1801133 SNP is located in the MTHFR catalytic region [13] [15]. A large number of studies have shown that MTHFR rs1801133 triggers anincrease in Hcy level by intervening the remethylation pathway of Hcy [12] [14]. Correlation between gene polymorphisms and childhood leukemia in different races and nations has been widely explored. However, their conclusions remain inconsistent [8] [9]. Our analysis suggested that MTHFR rs1801133 enhanced the risk of childhood leukemia, especially AG, GG or AG+GG allele. To our knowledge, this study for the first time evaluated the role of MTHFR rs1801133 in the pathogenesis of childhood leukemia.

Classified by genotypes of MTHFR rs1801133, we detected a higher susceptibility to childhood leukemia in people carrying GG or AG+GG allele in comparison to whom carrying AA allele. Moreover, people carrying G allele of MTHFR rs1801133 had a higher risk of suffering from childhood leukemia than whom carrying A allele. Potential influences of MTHFR rs1801133 polymorphism on clinical data of childhood leukemia cases and their chemotherapy response were further assessed. It is shown that higher risk of childhood leukemia may occur in people carrying AG+GG allele of MTHFR rs1801133 with a younger age (<15 years) or complete remission compared with people carrying AA allele of MTHFR rs1801133.

Three limitations in the present study main limit the reliability of our conclusion. First of all, a relatively small sample size wound affect the results. Secondly, potential influences of the internal environment of the embryo, nutritional intake of the mother during pregnancy or other important factors are not taken into considerations. Thirdly, a possible effect of genotypes carried by parents on the risk of their offspring remains unclear. Collectively, our finding requires to be further validated by the high-quality research involving a large sample size in multi-center hospitals.

## Conclusion

MTHFR rs1801133 gene polymorphism has a significant correlation with childhood leukemia. It is an important genetic susceptibility gene of childhood leukemia.

## Dodatak

### Conflict of interest statement

All the authors declare that they have no conflict of interest in this work.
